# Extracellular Matrix Remodeling and Matrix Metalloproteinases in Ovarian Function and Infertility

**DOI:** 10.3390/ijms27083652

**Published:** 2026-04-19

**Authors:** Efthalia Moustakli, Athanasios Zikopoulos, Periklis Katopodis, Vasilios Sebastian Paraschos, Ioannis Messinis, Christina Messini

**Affiliations:** 1 Department of Nursing, School of Health Sciences, University of Ioannina, 4th Kilometer National Highway Str. Ioannina-Athens, 45500 Ioannina, Greece; ef.moustakli@uoi.gr; 2 Department of Reproductive Medicine and Surgery, University College London Hospitals NHS Foundation Trust, 235 Euston Road, London NW1 2BU, UK; thanzik92@gmail.com; 3 Laboratory of Medical Genetics in Clinical Practice, Faculty of Medicine, School of Health Sciences, University of Ioannina, 45110 Ioannina, Greece; katopodisper@gmail.com; 4 Department of Obstetrics and Gynecology, Corewell Health Hospital, 4700 Schaefer Road Suite 310, Dearborn, MI 48126, USA; vasilispar10@gmail.com; 5 Department of Obstetrics and Gynaecology, Faculty of Medicine, School of Health Sciences, University of Thessaly, 41500 Larisa, Greece; messini@uth.gr

**Keywords:** oocyte maturation, extracellular matrix remodeling, oxidative stress, mitochondrial dysfunction, corpus luteum, folliculogenesis, luteal phase, assisted reproductive technology

## Abstract

Ovarian function relies on a network of well-coordinated molecular mechanisms that regulate follicular development, oocyte maturation, ovulation, and corpus luteum function. When these processes are disrupted, infertility can result. Extracellular matrix (ECM) remodeling represents a central regulatory component in these processes and is essential for follicle rupture and oocyte release. This mechanism involves metalloproteinases (MMPs), mainly MMP-2 and MMP-9, which degrade the ECM and allow the necessary structural changes. Other ECM-modulating proteases, such as ADAM and ADAMTS families, also contribute to this process. Their activity is tightly regulated by tissue inhibitors of metalloproteinases (TIMPs), ensuring that tissue remodeling occurs in a controlled manner. Disruption of the balance between MMPs and TIMPs increases the risk of infertility-related conditions such as polycystic ovary syndrome (PCOS), endometriosis, luteinizing hormone (LH) deficiency syndrome, and ovarian aging. In addition to the ECM, other factors, including intracellular signaling pathways, oxidative stress (OS), and mitochondrial function, contribute to ovarian physiology and directly affect oocyte quality and viability. This narrative review focuses on the molecular mechanisms governing ovarian function, with particular emphasis on the remodeling of the ECM by MMPs during ovulation, and examines how their disorders contribute to infertility. A deeper understanding of these mechanisms may lead to the identification of new therapeutic targets and the improvement of assisted reproduction outcomes.

## 1. Introduction

The ovary is a central regulator of female reproductive function, coordinating follicular development, hormone production, ovulation, and corpus luteum formation. These processes are controlled by highly integrated endocrine and molecular signaling networks, as well as dynamic structural changes within the ovarian microenvironment that ensure proper oocyte maturation and reproductive competence [1].

The highly synchronized ovarian cycle involves complex cellular and molecular interactions that enable oocyte growth and release for fertilization. Hormonal regulation through the hypothalamic–pituitary–ovarian (HPO) axis, including gonadotropins such as follicle stimulating hormone (FSH) and luteinizing hormone (LH), together with local ovarian factors such as growth factors and cytokines, governs follicle development from the primordial to the preovulatory stage. These processes are closely linked to extracellular matrix (ECM) remodeling, which provides structural support and regulates cell–cell and cell–matrix interactions. These coordinated processes are essential for progesterone production, corpus luteum formation, and successful ovulation [2].

Disruption of these regulatory mechanisms is observed in conditions such as polycystic ovary syndrome (PCOS) and premature ovarian insufficiency (POI). PCOS is associated with metabolic dysfunction, hyperandrogenism, and impaired follicular development, whereas POI is characterized by accelerated follicular depletion and increased risk of systemic complications, including osteoporosis and cardiovascular disease. A deeper understanding of ovarian molecular regulation is therefore critical for improving infertility management and optimizing assisted reproductive technologies (ART) [3,4].

Infertility affects a significant proportion of the global population, with ovarian dysfunction representing a major contributing factor. At the molecular level, infertility often arises from disruptions in key regulatory pathways that control folliculogenesis, oocyte maturation, ovulation, and luteal function. These disruptions may impair oocyte quality, hormonal balance, and follicular dynamics, ultimately reducing reproductive potential and ART success rates [5,6].

Emerging evidence indicates that ovarian function is governed by interconnected molecular pathways involving intracellular signaling cascades, oxidative stress (OS) responses, mitochondrial activity, and ECM remodeling. Among these, ECM remodeling is a key determinant of ovulation, as it enables structural changes required for follicular rupture [7,8,9]. This process is primarily mediated by metalloproteinases (MMPs), including multiple members of the MMP family, whose activity must be tightly regulated to ensure controlled tissue remodeling [10]. In addition, other ECM-modulating proteases, such as ADAM and ADAMTS families, contribute to the regulation of ovarian tissue dynamics.

Dysregulation of these pathways has been increasingly recognized as a central driver of infertility, linking alterations in intracellular signaling, OS, mitochondrial function, and ECM dynamics to impaired ovarian performance. In particular, disruption of the balance between MMPs and their inhibitors represents a critical mechanism underlying both physiological and pathological ovarian remodeling [11,12,13].

This review synthesizes current evidence on the molecular mechanisms regulating ovarian function, with a particular emphasis on ECM remodeling and MMP activity during ovulation. It further examines how disruption of these pathways contributes to infertility and highlights emerging molecular targets with potential relevance for improving reproductive outcomes.

## 2. Literature Search Strategy

This narrative review aims to synthesize the available data on the molecular mechanisms that regulate ovarian function, with a particular focus on the remodeling of the ECM, MMPs, and their role in infertility. The methodological approach is based on established principles for narrative reviews, integrating findings from different study designs to provide a comprehensive overview of key biological processes and clinical implications.

A structured literature search was conducted using the electronic databases PubMed, Scopus, and Web of Science. Search terms included combinations of keywords such as “ovarian function,” “folliculogenesis,” “oocyte maturation,” “ovulation,” “MMPs,” “ECM remodeling,” “infertility,” “oxidative stress (OS),” and “mitochondrial dysfunction”, as well as additional terms related to ECM-modulating proteases, including “ADAM,” “ADAMTS,” and “proteoglycanases.” Additionally, the bibliographic references of relevant articles were reviewed to identify additional studies.

Both primary and secondary sources were included. The primary studies involved experimental and clinical research on the molecular mechanisms underlying ovarian physiology and pathology. Secondary sources, including systematic and narrative reviews, were utilized to provide broader context and synthesize current knowledge.

The search focused primarily on studies published over the past two decades, reflecting significant advances in reproductive biology and molecular medicine. Earlier studies were included when they provided critical information on fundamental mechanisms.

The identified literature was evaluated for its relevance to the review’s objectives, prioritizing studies investigating molecular pathways related to follicular development, ovulation, ECM remodeling, ECM-related proteolytic enzymes, and infertility. The selected data were synthesized qualitatively, with an emphasis on mechanistic insights and their clinical relevance to reproductive disorders and ART.

## 3. Molecular Mechanisms in Ovarian Follicular Development

Follicular development is a tightly regulated process, and its disruption represents a major molecular basis of infertility. Dysregulation at any stage, from the activation of primordial follicles to the selection of the dominant follicle, can affect ovulation and reduce reproductive potential [14,15]. In addition to intracellular signaling pathways, these processes are influenced by dynamic changes in the ECM, which modulate the ovarian microenvironment and follicular architecture.

### 3.1. Primordial Follicle Activation

The migration of primordial follicles toward the developing pool constitutes the initial and highly regulated stage of folliculogenesis. This activation is primarily governed by the phosphatidylinositol-3-kinase (PI3K)/Akt and mechanistic target of rapamycin (mTOR) pathways, which regulate cell survival, proliferation, and metabolism [16,17]. These pathways maintain the balance between follicular activation and quiescence and are essential for the maintenance of ovarian reserve and ensuring reproductive longevity.

Dysfunction of these pathways can lead to premature depletion of the ovarian reserve, as seen in conditions such as POI and ovarian hyperstimulation syndrome [18].

Bidirectional signaling between theca cells and granulosa cells is critical for communication between the oocyte and surrounding somatic cells during the activation of primordial follicles [19]. Factors secreted by the oocyte, including bone morphogenetic proteins (BMPs) and growth differentiation factor 9 (GDF-9), regulate the differentiation, proliferation, and steroidogenesis of granulosa cells, promoting follicle development and enhancing their response to gonadotropins [20,21].

In contrast, anti-Müllerian hormone (AMH) and the forkhead box protein O3 (FOXO3) act as key inhibitory regulators, preserving ovarian reserve by inhibiting the premature activation of follicles. AMH, secreted by the granulosa cells of developing follicles, inhibits the recruitment of primordial follicles, while FOXO3 maintains their quiescence by suppressing PI3K/Akt signaling [22,23]. Although AMH and FOXO3 act through distinct regulatory mechanisms, both converge functionally on the suppression of primordial follicle activation, primarily via modulation of PI3K/Akt signaling, and no direct molecular interaction between them has been clearly established.

Disruptions in these regulatory mechanisms contribute to the development of reproductive disorders. In POI, excessive activation of primordial follicles accelerates the depletion of the follicular reserve, leading to infertility [24].

A thorough understanding of the molecular mechanisms governing the activation of primordial follicles is critical for optimizing ART and developing targeted fertility preservation strategies. Modifying these pathways represents a promising approach for preserving ovarian reserve and improving reproductive outcomes [16,25]. Emerging evidence suggests that ECM composition and remodeling also influence primordial follicle activation by regulating tissue stiffness, cell–matrix interactions, and the accessibility of growth factors, highlighting the role of ECM dynamics in early folliculogenesis. MMPs, although less active at this early stage compared to ovulation, contribute to controlled ECM turnover, thereby supporting follicular activation and microenvironmental remodeling.

### 3.2. Follicular Growth and Selection

Activated follicles undergo a complex sequence of growth and selection processes, which ultimately lead to the formation of a dominant follicle capable of ovulation. This tightly regulated process is controlled by both endocrine and paracrine signaling pathways and ensures that only a limited number of follicles reach full maturity, while the rest undergo atresia [26,27]. Disorders of these mechanisms are a major cause of anovulation and infertility.

Several growth factors, including GDF-9, insulin-like growth factors (IGFs), and BMPs, play a central role in the survival, differentiation, and proliferation of granulosa cells. BMP-15 promotes steroidogenesis and follicular development, while GDF-9 enhances the response of granulosa cells to gonadotropins. IGFs increase follicular sensitivity to gonadotropins and facilitate estrogen production by increasing the expression of the FSH receptor (FSHR) [21,28].

FSH and LH are the primary endocrine regulators of follicular development, acting through their respective receptors to control steroidogenesis. FSH stimulates aromatase activity in granulosa cells, promoting the conversion of androgens to estrogens, while LH induces androgen production in theca cells. The coordinated interaction between these cell types is essential for adequate estrogen synthesis, which supports follicular maturation and endometrial preparation [14].

During the follicular recruitment process, a dominant follicle is selected through competition for available FSH. The follicle with the highest estrogen production and the highest expression of the FSH receptor becomes dominant, while the decrease in FSH levels leads the remaining follicles to apoptosis and ultimately to atresia [29,30,31].

AMH also contributes to follicle selection by inhibiting excessive recruitment from the pool of primordial follicles and modulating their FSH-dependent development. Disruptions in AMH signaling are closely associated with infertility, particularly in PCOS, where elevated levels of AMH contribute to reduced follicular selection and anovulation [32,33].

According to Dewailly, dysfunction of the mechanisms governing follicle development and selection is a key feature of PCOS, contributing to anovulation, hormonal imbalance, and excessive follicular recruitment. These disorders affect not only ovulation but also follicle quality [34]. A deeper understanding of these mechanisms is critical for improving fertility treatments and developing personalized therapeutic strategies aimed at optimizing the outcomes of ART [35]. In parallel, ECM remodeling contributes to follicular growth and selection by regulating granulosa cell proliferation, migration, and structural organization, with proteolytic enzymes such as MMPs facilitating controlled matrix turnover during follicular expansion, thereby supporting granulosa cell function and follicular structural remodeling.

## 4. Oocyte Maturation and Molecular Regulation

Following follicular development, oocyte maturation is a critical process that determines developmental competence and fertilization potential. This process, which includes nuclear and cytoplasmic maturation, is carefully regulated by hormones, signaling molecules, and follicular cells within a dynamically remodeled ECM microenvironment [36]. Disruptions in these processes impair oocyte quality and developmental potential. In this context, MMPs contribute to ECM remodeling within the follicular microenvironment, facilitating structural and biochemical changes required for oocyte maturation.

### 4.1. Nuclear Maturation

Meiosis is arrested at prophase I at the germinal vesicle (GV) stage until triggered by ovulatory cues and resumes during nuclear maturation. This process depends on the maturation-promoting factor (MPF), primarily regulated by the cyclin B and cyclin-dependent kinase 1 (CDK1) complex [37]. Since Wee1/Myt1 kinases block cyclin-dependent kinase 1 (CDK1) complex. During prophase I, MPF remains inactive due to inhibitory phosphorylation mediated by Wee1/Myt1 kinases. Activation of Cdc25 phosphatase removes this inhibition, leading to MPF activation, chromosomal condensation, germinal vesicle breakdown (GVBD), and progression to metaphase II, where the oocyte remains arrested until fertilization [38,39].

MOS, a serine/threonine kinase, activates the MAPK pathway, regulating spindle dynamics and chromatin condensation. The MOS-MAPK cascade maintains meiotic spindle integrity and prevents premature chromosomal segregation [40]. These pathways can lead to aneuploidy, a major cause of infertility and embryonic developmental failure, particularly in assisted reproductive technologies (ART) [41]

High intracellular cAMP levels maintain meiotic arrest prior to the LH surge through activation of protein kinase A (PKA), which inhibits MPF activation. The LH surge reduces natriuretic peptide precursor C (NPPC) signaling via its receptor NPR2, leading to decreased cAMP levels and initiation of meiotic resumption [42,43]. Although nuclear maturation is primarily regulated by intracellular signaling pathways, ECM-mediated cues from surrounding follicular cells contribute indirectly by modulating cell–cell communication and the mechanical microenvironment of the oocyte.

### 4.2. Cytoplasmic Maturation

According to Watson (2007), cytoplasmic maturation is essential for ensuring that the oocyte possesses the molecular and metabolic components required to support early embryonic development [44]. This includes mRNA storage, organelle redistribution, and metabolic adaptation. Mitochondria play a central role as the primary source of ATP required for meiosis, fertilization, and early embryogenesis [45].

Mature oocytes exhibit a uniform mitochondrial distribution, supporting processes such as chromatin remodeling and spindle assembly. However, age-related mitochondrial dysfunction can lead to mtDNA mutations, increased ROS, and reduced ATP production, contributing to poor oocyte quality and embryonic arrest [9,46]. Impaired mitochondrial function plays a key role in fertility decline and affects ART. These alterations are closely associated with reduced fertilization rates and impaired ART outcomes [47].

Additionally, the stability and translation of stored maternal mRNAs are regulated by RNA-binding proteins, including cytoplasmic polyadenylation element-binding protein (CPEB) [48]. According to Piqué et al. (2008) [49], these transcripts are essential for early embryonic development, and their dysregulation compromises developmental competence. Defective mRNA regulation further compromises oocyte competence and is associated with failed embryogenesis [49]. These processes are also influenced by ECM remodeling, which regulates nutrient availability, cellular organization, and metabolic exchange within the follicular microenvironment.

### 4.3. Role of Cumulus Cells in Oocyte Maturation

Cumulus cells mediate essential metabolic and paracrine interactions with the oocyte, supporting its growth and developmental competence. These specialized granulosa cells provide vital nutrients such as lipids, nucleotides, and amino acids [50,51].

A critical function of cumulus cells is the regulation of energy metabolism. They provide glycolytic intermediates such as lactate and pyruvate, which are utilized by the oocyte due to its limited glycolytic capacity. Gap junctions between cumulus cells and the oocyte enable the transfer of small molecules, including cAMP, thereby maintaining meiotic arrest until maturation is initiated [52,53,54].

In response to the LH surge, cumulus cells release epidermal growth factor (EGF)-like peptides (AREG, EREG, and BTC), which activate the EGFR pathway [55]. This signaling cascade promotes oocyte maturation and developmental competence. Disruption of cumulus–oocyte communication is associated with reduced oocyte quality, impaired developmental potential, and poor ART outcomes [50,56]. ECM components and their remodeling enzymes further regulate cumulus–oocyte interactions by controlling the structural integrity and expansion of the cumulus matrix during oocyte maturation.

### 4.4. Hormonal Regulation of Oocyte Maturation

Oocyte maturation is coordinated by hormonal signals acting on the follicle–oocyte complex to trigger meiotic resumption and cytoplasmic maturation [57].

FSH prepares the oocyte for maturation by promoting cumulus expansion, metabolic activity, and increased responsiveness to LH, primarily through upregulation of the FSH receptor (FSHR) and metabolic gene expression. The preovulatory LH surge is the key trigger for oocyte maturation, including MPF activation and GVBD through reduction in intra-oocyte cAMP levels. LH also stimulates progesterone production by cumulus cells, further supporting cytoplasmic maturation and acquisition of developmental competence [55,58,59].

Estrogen contributes by regulating the expression of genes involved in follicular development and meiotic progression. Disruptions in hormonal regulation, as observed in PCOS and POI, impair oocyte maturation and reduce fertility [60]. Hormonal imbalance, including altered LH/FSH ratio, hyperandrogenism, and impaired estrogen and progesterone production, therefore represents a central molecular mechanism underlying infertility, particularly in conditions associated with anovulation and reduced oocyte quality [55,61].

These alterations reduce fertilization efficiency and early embryonic development. Hormonal signaling is closely linked to ECM remodeling, as gonadotropins regulate the expression of proteolytic enzymes, including MMPs, which facilitate structural changes required for oocyte maturation and ovulation. These processes are closely interconnected with intracellular signaling pathways, OS, and mitochondrial function, which collectively regulate oocyte quality and developmental competence within the ovarian microenvironment. The key molecular mechanisms regulating oocyte maturation and their associated dysfunctions are summarized in Table 1.

## 5. Ovulation and the Role of MMPs

**Figure 1:**
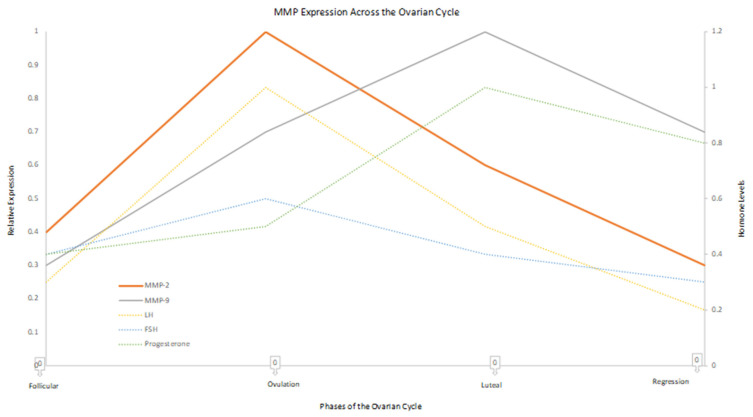
MMP expression and hormonal dynamics across the ovarian cycle. Relative changes in MMP-2 and MMP-9 expression across the follicular, ovulatory, luteal, and regression phases in relation to key hormonal fluctuations (LH, FSH, and progesterone). These coordinated changes highlight the role of MMPs in ECM remodeling and ovulation, while their dysregulation may contribute to infertility. This figure was created by the authors using Microsoft Excel.

Ovulation is a critical event in reproduction, and its disruption represents a major cause of infertility. Molecular mechanisms regulating ECM remodeling, particularly those involving MMPs, play a central role in follicular rupture and oocyte release as part of a broader network of ECM-modulating proteolytic enzymes [69,70]. The dynamic changes in MMP expression and their relationship with hormonal fluctuations across the ovarian cycle are illustrated in Figure 1.

### 5.1. Mechanisms Triggering Ovulation

The preovulatory LH surge initiates the highly coordinated process of ovulation, resulting in follicular rupture through a series of molecular and cellular events. LH activates intracellular signaling pathways mainly through cAMP and PKA following binding to G protein-coupled receptors on granulosa and theca cells [71]. This signaling cascade also activates downstream pathways, including PI3K/Akt and MAPK, which regulate granulosa cell differentiation, survival, and proteolytic activity, while enhancing the transcription of ovulation-related genes [72,73].

Localized prostaglandin synthesis is a key component of ovulation, mediating inflammatory and structural alterations within the follicle. The LH surge stimulates cyclooxygenase-2 (COX-2) expression in granulosa cells, increasing prostaglandin production. These mediators promote ECM remodeling and follicular wall destabilization by enhancing vascular permeability, leukocyte infiltration, and activation of proteolytic enzymes [26,71].

Ovulation is frequently characterized as an inflammatory-like process, involving the production of reactive oxygen species (ROS) and pro-inflammatory cytokines such as tumor necrosis factor α (TNF-α) and interleukins [74]. These mediators promote MMP activation, along with other ECM-modulating proteases, contributing to ECM degradation and facilitating follicular rupture (Table 2).

Despite its inflammatory characteristics, this process is tightly regulated to ensure controlled tissue remodeling without excessive damage. Effective oocyte release while maintaining ovarian tissue integrity is made possible by coordinated interactions among inflammatory mediators, proteolytic enzymes, and vascular alterations. Ovulatory dysfunction results from the disruption of these regulatory processes, which also affect follicular rupture and ECM remodeling. In diseases such as PCOS and luteinized unruptured follicle syndrome, these changes are crucial contributors to infertility [26,75].

**Table 2:** Key inflammatory mediators involved in ovulation and infertility. Summary of major inflammatory mediators, their sources, and roles in ECM remodeling and follicular rupture during ovulation.

Mediator	Source	Function	Effect on Ovulation	References
TNF-α	Granulosa cells; Theca cells	Induces inflammatory signaling and promotes ECM degradation	Facilitates follicular rupture through MMP activation and other proteolytic pathways; dysregulation may impair ovulation and contribute to infertility	[76,77]
IL-1β	Granulosa cells; Leukocytes	Stimulates MMP expression and amplifies local inflammatory responses	Promotes ECM remodeling and follicular wall weakening via activation of proteolytic enzymes; abnormal levels may disrupt ovulation	[78,79]
IL-6	Granulosa cells; Theca cells	Modulates immune responses and granulosa cell differentiation	Enhances MMP activity and ECM remodeling processes; dysregulation may impair ovulatory function	[80,81]

### 5.2. MMPs and Their Role in ECM Degradation and Ovulation

#### 5.2.1. Role of MMPs in ECM Degradation

ECM degradation is a critical step in ovulation, enabling follicular wall remodeling and oocyte release. MMPs, a family of zinc-dependent endopeptidases, degrade key ECM components including collagen, fibronectin, and laminin, thereby weakening the follicular matrix and facilitating rupture [82,83].

MMP-2 and MMP-9 are among the principal enzymes involved in this process, although additional members of the MMP family also contribute to ECM remodeling during ovulation. By targeting basement membrane components such as type IV collagen and laminin, these gelatinases destabilize the ECM and promote granulosa cell motility, enabling coordinated tissue remodeling during ovulation [84].

Beyond matrix degradation, MMPs contribute to broader ovarian remodeling processes, including angiogenesis and cellular reorganization, highlighting their multifunctional role in ovarian physiology [83].

Their activity is tightly regulated by tissue inhibitors of metalloproteinases (TIMPs), which prevent excessive proteolysis and ensure spatial and temporal control of ECM remodeling. Maintenance of the MMP–TIMP balance is essential for normal ovulation [85].

Disruption of this balance impairs follicular rupture and has been associated with infertility-related disorders, underscoring the importance of precise regulation of ECM remodeling (Table 3).

#### 5.2.2. Upregulation of MMPs in Response to LH Surge

The preovulatory LH surge induces MMP overexpression in granulosa and theca cells, initiating the proteolytic processes required for follicular rupture. LH receptor activation stimulates G protein-coupled signaling pathways, increasing intracellular cAMP levels and activating PKA, which promotes transcription of ovulation-related genes, including MMP-2 and MMP-9 as well as other MMP family members involved in ECM remodeling [26,72].

Nuclear factor kappa B (NF-κB) is a key mediator of this process, regulating the expression of inflammatory and proteolytic genes. Under baseline conditions, NF-κB is sequestered in the cytoplasm by inhibitory IκB proteins. Following LH stimulation, IκB undergoes phosphorylation and degradation, allowing NF-κB translocation to the nucleus and activation of MMP gene transcription and other protease-related genes [89].

NF-κB also interacts with other signaling pathways, such as mitogen-activated protein kinase (MAPK) and phosphoinositide 3-kinase (PI3K)/protein kinase B (Akt), coordinating cellular changes required for ovulation and further enhancing MMP expression and ECM remodeling processes [6].

In parallel, the Fas/FasL apoptotic system contributes to follicular remodeling. Activation of this pathway initiates the caspase cascade, leading to granulosa cell apoptosis and ECM degradation and facilitating follicular rupture [90].

TIMPs maintain tight regulation of MMP activity, ensuring controlled ECM degradation. Disruption of LH signaling, NF-κB activation, or the MMP–TIMP balance impairs follicular rupture and contributes to infertility (Table 4).

#### 5.2.3. Specific MMPs Involved in Ovulation and Cellular Changes

MMP-2 and MMP-9 are among the primary enzymes involved in ECM remodeling during ovulation, although additional MMP family members also contribute to these processes. By degrading basement membrane components such as type IV collagen and laminin, they promote structural changes required for follicular rupture and granulosa cell migration [83,98].

Although their functions overlap, these enzymes exhibit distinct substrate specificities. MMP-2 targets multiple collagen types, including types I, IV, and V, whereas MMP-9 primarily degrades type IV collagen and fibronectin. Their activity is tightly controlled by TIMPs, ensuring localized and regulated ECM remodeling [83].

Dysregulation of MMP activity is associated with several infertility-related conditions. Reduced MMP activity contributes to luteinized unruptured follicle syndrome, whereas increased TIMP expression in PCOS inhibits ECM degradation, leading to follicular arrest and anovulation [99]. Conversely, excessive MMP activity in endometriosis promotes abnormal tissue remodeling and may disrupt the follicular microenvironment [100].

Ovulation also involves coordinated cellular processes, including granulosa cell apoptosis and luteinization. Apoptosis is primarily mediated by the Fas/FasL signaling pathway, which activates the caspase cascade, including caspase-8 and caspase-3, leading to granulosa cell degradation and facilitating follicular rupture [72,90,101].

The Bcl-2 protein family further regulates apoptosis through mitochondrial pathways, where increased membrane permeability leads to cytochrome c release and caspase activation. These apoptotic mechanisms act in concert with MMP-mediated ECM remodeling to ensure efficient follicular rupture [102].

Simultaneously, LH-induced luteinization transforms granulosa cells into progesterone-producing luteal cells, supporting the early luteal phase and endometrial preparation. The coordination of ECM remodeling, apoptosis, and luteinization is therefore essential for successful ovulation [103].

Disruption of this integrated network, involving MMP activity, TIMP regulation, and apoptotic signaling, impairs follicular rupture and contributes to infertility [104]. The relationship between MMP dysregulation and infertility-related conditions is summarized in Table 5.

Alterations in other ECM-modulating proteases may also contribute to these conditions but are not exhaustively represented in this table. Overall, dysregulation of the MMP–TIMP balance and ECM composition plays a central role, with reduced MMP activity contributing to ECM accumulation and follicular arrest in PCOS and POI, whereas excessive MMP activity promotes pathological tissue remodeling in endometriosis. Modulation of these pathways has been suggested as a potential strategy to improve ovarian function and fertility outcomes.

#### 5.2.4. Broader Spectrum of ECV Remodeling Enzymes in Ovarian Function

While MMP-2 and MMP-9 are among the most extensively studied enzymes in ovarian ECM remodeling, accumulating evidence indicates that a broader spectrum of MMP family members contributes to ovarian physiology and pathology [99,112].

MMP-3 (stromelysin-1) plays an important role in degrading proteoglycans and activating other MMPs, thereby amplifying proteolytic cascades during ovulation. MMP-7 is involved in epithelial remodeling, whereas MMP-8 and MMP-12 are associated with inflammatory processes that characterize ovulation as an inflammation-like event. In addition, membrane-type MMPs, particularly MMP-14, regulate pericellular proteolysis and facilitate granulosa cell migration, angiogenesis, and follicular restructuring [112,113].

In addition to MMPs, other ECM-modulating protease families, including a disintegrin and metalloproteinases (ADAM) and a disintegrin and metalloproteinases with thrombospondin motifs (ADAMTS), contribute to ovarian ECM dynamics. ADAMTS proteoglycanases are involved in the degradation of ECM components such as versican, thereby supporting follicular expansion and rupture. Notably, reduced expression of ADAMTS-1, ADAMTS-4, ADAMTS-5, and ADAMTS-9 has been associated with impaired in vitro fertilization outcomes in women with polycystic ovary syndrome [84,114].

These findings highlight that ovarian ECM remodeling is governed by a complex and coordinated network of proteolytic enzymes, rather than a limited subset of MMPs, underscoring the importance of considering multiple enzyme families in the context of ovarian physiology and infertility [99].

### 5.3. The Balance of MMPs and TIMPs

The activity of MMPs during ovulation is tightly controlled by TIMPs, which regulate the extent and timing of proteolytic activity within the follicular environment. To facilitate follicular rupture without causing severe tissue damage, this regulatory interaction ensures that ECM remodeling occurs in a spatially and temporally coordinated manner within a broader network of ECM-modulating proteases [115].

Alterations in the MMP–TIMP equilibrium are closely associated with ovulatory dysfunction. In PCOS, increased TIMP expression suppresses proteolytic activity, contributing to follicular arrest and anovulation [116]. Conversely, excessive MMP activity observed in endometriosis promotes abnormal tissue remodeling and may disrupt the follicular microenvironment. Age-related changes in MMP and TIMP expression further impair ECM turnover, contributing to fibrosis and reduced ovarian function [117].

Beyond proteolysis, TIMPs are also involved in angiogenesis and cell survival. Through modulation of VEGF signaling, they contribute to vascular remodeling during ovulation and luteal development. These functions highlight their broader role in maintaining ovarian tissue homeostasis [83,103,117].

**Figure 2:**
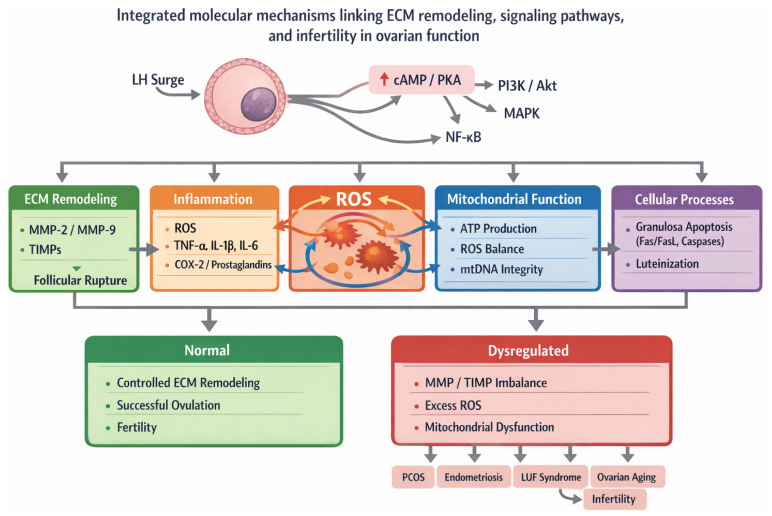
Integrated molecular mechanisms linking ECM remodeling, intracellular signaling, and infertility in ovarian function. The preovulatory LH surge activates multiple signaling pathways, including cAMP/PKA, PI3K/Akt, MAPK, and NF-κB, leading to coordinated regulation of inflammatory mediators, mitochondrial activity, and MMPs. These processes drive ECM remodeling and follicular rupture. Tight regulation by TIMPs ensures controlled proteolysis. Dysregulation of these interconnected pathways results in oxidative stress, mitochondrial dysfunction, and impaired ECM turnover, contributing to ovulatory disorders such as polycystic ovary syndrome, endometriosis, and ovarian aging, ultimately leading to infertility.

Disruption of this regulatory balance can impair follicular rupture and represents an important molecular mechanism contributing to infertility (Figure 2).

## 6. Post-Ovulation: Luteal Phase and Corpus Luteum Formation

Following ovulation, granulosa cells undergo luteinization under the influence of LH, differentiating into luteal cells that collectively form the corpus luteum (CL), a temporary endocrine structure responsible for progesterone production [118]. Progesterone supports endometrial remodeling, increasing uterine receptivity and facilitating early embryo implantation. Impaired luteal function, characterized by insufficient progesterone synthesis, is associated with reduced reproductive success and luteal phase deficiency [119].

During CL formation, extensive microvascular remodeling occurs alongside dynamic ECM remodeling. Angiogenesis is essential for luteal development, ensuring an adequate blood supply to sustain high steroidogenic activity. This process is primarily mediated by VEGF, which promotes endothelial cell proliferation and migration, and capillary formation. Disruption of angiogenic signaling can impair luteal development and compromise implantation [120].

### 6.1. LH’s Role in Corpus Luteum Maintenance

During the early luteal phase, LH is required to maintain CL function and prevent premature luteolysis. LH signaling sustains steroidogenic activity in luteal cells and inhibits apoptosis. It also stimulates the expression of steroidogenic acute regulatory (StAR) protein, which promotes cholesterol transport into mitochondria for progesterone synthesis [121]. Estradiol further enhances luteal responsiveness by modulating LH receptor sensitivity, supporting optimal progesterone production. Disruption of LH signaling impairs luteal function and reduces endometrial receptivity [122].

### 6.2. Human Chorionic Gonadotropin and Corpus Luteum Regression

Human chorionic gonadotropin (hCG), secreted by the developing embryo after fertilization, binds to luteal LH receptors to sustain CL activity. This signaling maintains progesterone synthesis until placental steroidogenesis is established. Exogenous progesterone or hCG is frequently utilized in ART to enhance implantation results and sustain luteal function [123,124].

The CL experiences luteolysis when there is no pregnancy. Decreased progesterone synthesis and the start of luteal regression are caused by reduced LH support. By interfering with luteal vascularization and stimulating inflammatory signals within the CL, prostaglandins, especially PGF2α, aid in this process [120].

MMPs, including MMP-2 and MMP-9, as well as other ECM-modulating proteases, mediate ECM remodeling during luteolysis. During this stage, their activity is tightly regulated to ensure controlled tissue destruction [83].

### 6.3. MMPs in Corpus Luteum Regression and Regulation

By weakening the corpus luteum’s structural elements, MMPs, along with other ECM-remodeling enzymes, aid in tissue reorganization during luteal regression. The collapse of luteal architecture and the transition to the subsequent ovarian cycle are supported by this mechanism. TIMPs modulate MMP activity, ensuring regulated proteolysis during CL involution [84,125].

Impaired luteal regression and luteal phase abnormalities have been linked to altered control of MMP activity. Therefore, modifying luteal function in reproductive disorders may be possible by focusing on MMP-mediated pathways [121]. These processes further highlight that luteal regression is governed by a coordinated network of proteolytic systems rather than isolated enzymatic activity.

## 7. Ovarian Aging and Implications for Reproductive Health

Ovarian aging is characterized by a progressive decline in ovarian reserve and oocyte quality. This process eventually decreases reproductive potential by causing the progressive loss of primordial follicles and functional degradation of remaining oocytes [126]. Estradiol synthesis is hampered, and follicular growth is disrupted by age-related changes in hormonal signaling, specifically decreased reactivity to FSH [127].

These alterations are caused by a confluence of environmental, hormonal, and genetic variables that impact important regulatory mechanisms related to ovulation and folliculogenesis [128]. Additionally, by raising OS and upsetting hormonal homeostasis, lifestyle and environmental exposures, such as smoking and endocrine-disrupting substances, hasten ovarian aging. All of these changes have a detrimental effect on fertility and lower the effectiveness of ART [129].

### 7.1. Molecular Mechanisms of Ovarian Aging: OS, Mitochondrial Dysfunction, and ECM Remodeling

Ovarian aging is associated with interconnected molecular processes, including OS, mitochondrial dysfunction, and altered ECM remodeling within a complex proteolytic microenvironment [130].

OS leads to the accumulation of reactive oxygen species (ROS), which damages lipids, proteins, and DNA, compromising oocyte integrity and developmental competence [11]. ROS also modulate MMP activity through redox-sensitive signaling pathways, including NF-κB and MAPK, thereby linking mitochondrial dysfunction to ECM remodeling and ovarian aging. Mitochondrial dysfunction further exacerbates this process by reducing ATP production and disrupting cellular metabolism. Age-related accumulation of mitochondrial biogenesis contributes to reduced oocyte quality, increased aneuploidy, and poor reproductive oocytes [47].

Alterations in ECM remodeling also play a significant role in ovarian aging. Dysregulation of MMP activity and other ECM-modulating proteases disrupts follicular architecture and tissue turnover. Reduced MMP activity promotes fibrosis through the accumulation of ECM proteins, whereas excessive MMP activity promotes degradation of structural components [10,131]. Age-related changes in the MMP–TIMP balance exacerbate fibrotic remodeling, which hinders ovarian function and follicular growth. Inflammatory mediators such as TGF-β and IL-6 promote ECM deposition and tissue remodeling, thereby contributing to these processes [132].

### 7.2. Systemic Impact and Therapeutic Approaches

Decreased ovarian function has systemic effects that extend beyond reproduction, primarily due to lower levels of estrogen and progesterone. Reduced bone density, increased cardiovascular risk, and potential neurocognitive decline are associated with estrogen deficiency [133]. These effects highlight that ovarian aging broadly affects the body’s physiology.

Various approaches are being explored to address age-related decline in ovarian function. Resveratrol and coenzyme Q10 are antioxidants that enhance mitochondrial function and reduce OS [134], and may indirectly influence MMP activity through modulation of redox-sensitive signaling pathways, although direct evidence in ovarian tissue remains limited. Modulation of MMP activity, therapies targeting mitochondrial function, as well as regenerative techniques, including ovarian tissue transplantation and stem cell therapy, are emerging therapeutic approaches [135]. Furthermore, lifestyle interventions, such as calorie restriction and regular exercise, can improve mitochondrial performance and reduce oxidative damage [136].

Fertility preservation techniques, such as oocyte cryopreservation, help enhance reproductive potential. Although their application requires careful individualization, HRT is utilized in clinical practice to address the systemic effects of estrogen deficiency [137].

## 8. Conclusions

Ovulation, luteal function, and follicular development are regulated by complex, interrelated molecular processes. Among these, follicular rupture and tissue remodeling depend largely on the dynamics of the ECM, which functions as an integrative regulatory framework and is primarily driven by MMPs along with other ECM-modulating proteolytic enzymes. Maintaining ovarian homeostasis requires strict control of MMP activity, particularly through their interaction with TIMPs.

Disruption of these mechanisms contributes to ovulatory dysfunction and infertility, as observed in conditions such as PCOS, endometriosis, luteal phase defects, and ovarian aging. These findings highlight the importance of coordinated signaling and tightly regulated proteolytic activity in reproductive physiology.

Targeting the molecular pathways involved in ECM remodeling, mitochondrial function, and OS represents a promising approach for improving fertility. In particular, modulating MMP activity and other ECM-related proteolytic systems, including ADAM and ADAMTS families, may offer new therapeutic approaches for optimizing ART and restoring ovulatory function.

Further investigation of the interaction between signaling pathways, tissue remodeling, and cellular metabolism in the ovary within this integrated ECM-centered network is expected to enhance our understanding of reproductive biology and contribute to the development of targeted interventions for infertility. These insights have direct clinical relevance, as dysregulation of ECM remodeling and MMP activity contributes to infertility-related conditions such as PCOS, endometriosis, and ovarian aging. Targeting these molecular pathways may improve diagnostic approaches and enhance outcomes in ART.

## Figures and Tables

**Table 1:** Molecular regulation of oocyte maturation and associated mechanisms contributing to infertility.

Maturation Type	Key Events	Regulatory Factors	Potential Dysfunction	References
Nuclear Maturation	Resumption of meiosis; GVBD; Chromosome condensation; Metaphase II arrest	MPF (CDK1 + Cyclin B); MOS-MAPK; Cdc25; cAMP/PKA; ECM microenvironment	Aneuploidy; Meiotic arrest; Implantation failure and infertility	[36,62]
Cytoplasmic Maturation	Organelle redistribution; Mitochondrial positioning; Accumulation of mRNAs & proteins	Mitochondrial activity; CPEB; metabolic adaptation; ECM remodeling	Reduced ATP production; Poor oocyte quality; Embryonic arrest; Reduced fertilization and ART success	[63,64]
Role of Cumulus Cells	Metabolic & paracrine support; Nutrient transfer; Gap junction communication; Regulation of oocyte microenvironment	Glycolysis and metabolite transfer (pyruvate, lactate); EGF-like peptides (AREG, EREG, BTC); cAMP signaling; cumulus ECM expansion and remodeling	Poor oocyte energy metabolism; Lower developmental competence; Reduced oocyte quality; Infertility	[65,66]
Hormonal Regulation	Meiotic resumption; Cumulus expansion; Follicular synchronization	FSH and FSH receptor (FSHR); LH surge (cAMP reduction); estrogen and progesterone signaling; MMP-mediated ECM remodeling	PCOS; POI; Anovulation; Infertility	[67,68]

**Table 3:** Representative MMPs involved in ECM remodeling during follicular rupture, their targets, and regulatory mechanisms.

MMP	Role in Ovulation	ECM Targets	Regulation	References
MMP-2	Degrades basement membrane components to facilitate follicular rupture and oocyte release	Type IV collagen; Fibronectin; Laminin	Activated by LH surge; Regulated by TIMPs	[82,85]
MMP-9	Promotes ECM degradation and granulosa cell migration during follicular rupture	Type IV collagen; Laminin; Elastin	Upregulated by LH surge; NF-κΒ signaling	[70,86]
MMP-1	Degrades interstitial collagen to support ECM remodeling during ovulation	Type I collagen	Regulated by TIMPs during follicular rupture	[87]
MMP-3	Degrades proteoglycans and activates other MMPs, amplifying proteolytic cascades	Proteoglycans; Aggrecan	Induced by inflammatory mediators	[88]

**Table 4:** Signaling pathways regulating ovulation, MMP activity, and infertility. Key pathways and molecules involved in ECM remodeling, granulosa cell function, and follicular rupture.

Pathway	Key Molecules	Function	Outcome	References
cAMP/PKA	cAMP; PKA	Induces transcription of ovulation-related genes, including MMPs	MMP upregulation and activation of ECM remodeling processes leading to ECM degradation and follicular rupture	[72,91]
NF-κΒ	NF-κΒ; IκΒ proteins	Regulates transcription of inflammatory and MMP-related genes	Increased MMP production and broader proteolytic activity, ECM remodeling during ovulation	[92,93]
MAPK	MAPK; ERK	Controls granulosa cell differentiation and ovulatory signaling	Enhanced MMP expression and ECM remodeling processes, enabling cellular changes required for follicular rupture	[6,94]
PI3K-Akt	PI3K; Akt	Promotes cell survival, proliferation, and angiogenic signaling	Supports granulosa cell survival, angiogenesis, and follicular development	[95,96]
Fas/FasL	Fas; FasL; caspase cascade (e.g., caspase-3,-8)	Induces apoptosis in granulosa cells	Granulosa cell apoptosis, ECM degradation, and facilitation of follicular rupture	[97]

**Table 5:** Overview of how altered MMP activity affects ovulation and contributes to infertility-related conditions.

Condition	MMP Dysregulation	Effect on Ovulation/Fertility	References
PCOS	Increased TIMP expression and reduced MMP activity, resulting in impaired ECM degradation	Follicular arrest, failure of follicular rupture, anovulation, and infertility	[105,106]
Endometriosis	Elevated MMP activity causing excessive ECM remodeling and tissue invasion	Disrupted follicular environment and reduced fertility	[107,108]
Luteinized Unruptured Follicle Syndrome	Impaired MMP activation or dysregulated proteolytic activity	Failure of follicular rupture and ovulatory dysfunction	[109]
Ovarian Aging	Imbalance between MMPs and TIMPs, often associated with fibrosis and altered ECM turnover	Impaired follicular remodeling, reduced ovulatory efficiency, and age-related infertility	[110,111]

## Data Availability

No new data were created or analyzed in this study.
